# Effect of edible film prepared with plasma‐activated water and olive leaf extract (*Olea europaea* L.) as a potential packaging in cooked meat product

**DOI:** 10.1002/fsn3.4482

**Published:** 2024-09-30

**Authors:** Damla Bilecen Şen, Ali Güleç

**Affiliations:** ^1^ Department of Food Engineering, Faculty of Engineering and Architecture Burdur Mehmet Akif Ersoy University Burdur Turkey; ^2^ Department of Biomedical Engineering, Faculty of Technology Isparta University of Applied Sciences Isparta Turkey

**Keywords:** edible film, meat and meat products, olive leaf extract, plasma‐activated water

## Abstract

This research determined the use of edible film (EF) prepared with olive leaf extract (OLE) and plasma‐activated water (PAW) on the shelf life and quality of cooked meat (*M. longissimus thoracis* et *lumborum*) product. The characterization of PAW composition and the antioxidant activity and the total phenolic content (TPC) of OLE were determined. Also, physicochemical and microbiological properties and lipid oxidation of cooked meat product coated with EF stored at +4°C for 14 d were evaluated. Cold plasma treatment increased the nitrite/nitrate concentrations and ORP value and decreased the pH value of distilled water (DW). The amount of TPC, IC_50_ value, and the antioxidant activity of OLE were 71.52 mg GAE/g, 0.46 mg/mL, and 77.39%, respectively. The combined use of OLE and PAW in EF during storage reduced the pH of cooked meat product. Furthermore, the addition of OLE in EF increased the b* values and reduced the a* values of cooked meat product, whereas the addition of PAW did not influence the CIE L*, a*, b* color values. The use of EF prevented lipid oxidation in cooked meat product during storage, especially the addition of OLE to the EF reduced the TBARS values. In this research, the addition of OLE to EF prepared with PAW has a promising application potential in the preservation of RTE‐cooked meat products. Also, these findings suggested that OLE, a by‐product from production of olive oil, might be applied as active packaging in biodegradable films.

## Graphical abstract

The use of edible film (EF) prepared with olive leaf extract (OLE) and plasma‐activated water (PAW) on the shelf life and quality of cooked meat product was evaluated. The combined use of OLE and PAW in EF reduced the pH and a* values, increased the b* values, and prevented the lipid oxidation in cooked meat product, with OLE specifically reducing TBARS values. Adding OLE to EF prepared with PAW has promising potential for preserving RTE‐cooked meat products.

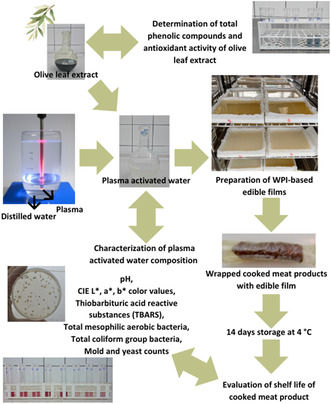

## 1 INTRODUCTION

Cold plasma (CP) treatment, a non‐thermal technology, is a versatile and emerging application with several uses in the food industry (Jadhav & Annapure, 2021). The CP, generated by the ionization of gases such as argon, helium, nitrogen, and oxygen in the atmospheric pressure or vacuum, is defined as a partially ionized gas containing molecules, free radicals, positive and negative ions, electrons, ultraviolet photons, and visible light (Oh et al., 2016). These plasma species may interact with the cells of bacterial and efficiently make a variety of microorganisms, including viruses and spores, inactive (Misra et al., 2011). This technology has recently been used to activate liquids like water by exposing them to CP discharges, which produces a mix of reactive oxygen and nitrogen species (RONS). The concentration and type of reactive species in plasma‐activated water (PAW) can lower the pH while increasing the conductivity and oxidation–reduction potential (ORP) of the solution, depending on operational variables such as the plasma power, CP source configuration, and activation duration (Risa Vaka et al., 2019). PAW is categorized as purified water that might be applied in the food industry rather than as a natural resource or chemical reagent (Thirumdas et al., 2018). The main factor in the reported PAW preservation effectiveness has been identified as the combination action of RONS, low pH, and high ORP. Furthermore, the discharge of CP above or below the surface of water frequently results in the production of PAW, which generates secondary reactive species like the nitrite (NO_2_^−^), nitrate (NO_3_^−^), hydroxyl radical (OH), hydrogen peroxide (H_2_O_2_), singlet oxygen, superoxide, and ozone (Zhao et al., 2018). However, the biggest problem with using PAW or CP discharge in fresh foods (unprocessed or minimally processed products stored in the refrigerator) is that it encourages protein and lipid oxidation, which lowers the acceptability of the end product (Olatunde et al., 2020). Antioxidants may have helped to prevent oxidation to solve this problem. Gao et al. (2021) reported that the combination action of dielectric barrier‐discharge CP and natural plant extracts (such as pine bark, rosemary, and pomegranate) increase the shelf life of chicken breast meat patties while retarding of lipid oxidation compared to patties treated with CP alone.

The improvement of biodegradable food packaging films based on renewable resources has received a lot of attention recently. Proteins, polysaccharides, and lipids are widely investigated materials for food packaging (Cazon et al., 2017; Chen et al., 2019; Milani & Nemati, 2022). Among these, protein‐based materials like whey protein isolate were a significant research area through latest decades due to excellent mechanical stability and gas barrier property (Mellinas et al., 2016). To extend the shelf life of food, edible films can also act as transporters for antioxidant and antibacterial compounds (Acevedo‐Fani et al., 2015). The addition of bioactive compounds to packaging materials has advantages over direct food addition, such as a lesser number of active compounds required, regulated release to food, and the removal of unnecessary processing process (Ramos et al., 2014). In this sense, olive leaf extract (OLE), which has been detected to be a potent antioxidant in reducing or eliminating lipid oxidation, is a natural compound (Hayes et al., 2009). The olive oil production generates significant quantities of by‐products, including crude olive cake, vegetative water, leaves, and twigs (10% of total olive weight). Olive leaf is another by‐product of olive grove farming that accumulates after olive tree (*Olea europea* L.) trimming (Guinda et al., 2004). Although olive leaves and cakes are frequently feed for animals, they also have greater added value uses in the cosmetic, pharmaceutical, and food sectors (Moudache et al., 2016). The antioxidant activity of olive leaf and cake extracts are attributable to phenolic components such as oleuropein, hydroxytyrosol, and luteolin (Botsoglou et al., 2014; Bouaziz et al., 2008). These bioactive substances can be included in edible films or coatings to improve their properties, such as antimicrobial and antioxidant activity. Previous studies demonstrated that the strong antioxidant activity of active films in which OLE was incorporated (Licciardello et al., 2015; Marcos et al., 2014; Martiny, Pacheco, et al., 2020; Moudache et al., 2016). In addition, Moudache et al. (2017) and Ozvural (2019) reported that edible films containing OLE decreased the lipid oxidation and extended the shelf life of fresh pork meat and chicken nuggets, respectively. Moreover, Martiny, Raghavan, et al. (2020) revealed that the edible film with the OLE demonstrated an antibacterial capacity during the storage of lamb meat during the storage period, resulting in a fivefold decrease in the count of psychrophilic microorganisms as compared to control and samples packaged in commercial films.

The shelf life and oxidative stability of ready‐to‐eat (RTE) meat products, such as döner kebab, meatball, and hamburger patty, are vital for the meat industry to address consumer demands for both health and quality. Lipid oxidation and microbial spoilage are recognized as major factors affecting the quality of RTE meat products (Falowo et al., 2014). Quality deterioration or healthy risks caused by oxidation reactions or microbial spoilage significantly worsen during storage, resulting in decreased marketability and consumer acceptance of RTE meat products. For this reason, a novel approach for increasing the shelf life of meat products is still necessary. Thus, this study aimed to determine the addition of OLE to edible film prepared with PAW on the shelf life and quality of cooked meat product stored at +4°C for 14 d. PAW reduces the microbial load in cooked meat product, while the edible film acts as an oxygen barrier to restrict oxygen involvement in the oxidation process generated by the PAW treatment. The phenolic compounds of OLE may also retard the lipid oxidation by acting as a free radical scavenger and prevent the growth of spoilage microorganisms during the storage period. The impacts of edible film, OLE addition, PAW treatment, and combining of all these applications on the shelf life and quality of cooked meat product were determined. The findings can help increase economic competitiveness by improving the quality and safety of RTE meat products.

## 2 MATERIALS AND METHODS

### 2.1 Materials

Whey protein isolate (100% WPI, BiPRO, Davisco Foods International Inc., USA) and olive leaf extract (OLE) were provided from Hardline Nutrition (İstanbul, TR) and Immunat Bitkisel Ilac (Muğla, TR), respectively. Analytical grade chemicals were used in whole analysis.

### 2.2 Preparation of plasma‐activated water (PAW)

PAW was prepared by a water–air discharge system at atmospheric pressure. The stainless‐steel rod was placed inside the quartz tube and connected to the kHz power source. A grounded aluminum electrode was placed outside the tube. Air was injected into the reactor at a flow rate of 50 L/min with 2.4 L/min water. 190 W was delivered to the inner electrode, filamentary discharge occurred around the inner electrode's tip. Following a 10‐min discharge, PAW with H_2_O_2_ concentrations of 100 ppm were measured using MQuant Peroxide Test strips (Merck, Darmstadt, DE).

### 2.3 Characterization of PAW composition

The concentration of some RONS found in the PAW was determined (Table 1). Ion chromatography (Dionex ICS‐3000 Ion Chromatograph (Sunnyvale, CA) equipped with a Dionex GM‐4 gradient mixer, a conductivity detector, and Dionex AS9‐HC 4 mm and AG9‐HC 4 mm columns) was used for NO_2_^−^ and NO_3_^−^ analyses according to Standard Method 4110 B (APHA, 1998). pH and ORP values (HI 2211 pH/ORP Meter, Hanna Instruments, Woonsocket, RI, USA) in the PAW were also characterized.

**TABLE 1:** Concentration of nitrites (NO_2_^−^), nitrates (NO_3_^−^), and hydrogen peroxide (H_2_O_2_), pH and ORP in distilled water (DW), and plasma‐activated water (PAW).

	NO_2_^−^(mg/L)	NO_3_^−^ (mg/L)	H_2_O_2_ (mg/L)	pH	ORP (mv)
DW	ND	ND	ND	8.00 ± 0.1	−43.5 ± 1.2
PAW	6.98 ± 0.72	5.79 ± 0.35	100	5.80 ± 0.1	64.4 ± 1.98

Abbreviation: ND, not detected.

### 2.4 Determination of total phenolic compounds (TPC) and antioxidant activity of olive leaf extract (OLE)

TPC in OLE was analyzed by the Folin‐Cicalteu method (Singleton & Rossi, 1965). TPC was quantified as the equivalent of gallic acid (GAE) in mg/g extracts using a standard curve prepared with varied gallic acid concentrations.

The 1,1‐diphenyl 2‐picrylhydrazyl (DPPH) radical scavenging test was analyzed according to Dorman et al. (2003). The percentage of DPPH radical inhibition is used to represent the antioxidant activity of OLE extract. Also, a graph of the inhibition percentage versus concentration was used to calculate the half‐maximum inhibition concentration (IC_50_) for DPPH radical scavenging.

The ferric‐reducing antioxidant power (FRAP) test was analyzed according to Benzie and Strain (1996). The results were represented as μmol Fe^+2^/L. A standard curve was generated with Fe^+2^ values ranging from 100 to 2000 μmol/L.

### 2.5 Preparation of WPI‐based edible films

The WPI‐based edible film was made according to Akcan et al. (2017). The coating solution was prepared by dissolving 5% (w/v) WPI in distilled water and mixing it with 5% (w/v) glycerol. The pH of the mixture was adjusted to 8.0 using 2 N NaOH. The solution was then heated to 90°C for approximately 30 min until it became uniform and was subsequently allowed to cool to room temperature. OLE extract was added to the WPI‐based edible film solution to achieve a final concentration of 0.5% (v/v), based on the weight of the solution. Then, the 5% (v/v) OLE extract was added to half of the WPI‐based solution. The solution was homogenized for 1 min with homogenizer (IKA T25, DE). Table 2 shows the film solution composition and the related sample codes utilized during the study. The 50 mL prepared solution was cast into sterile polyethylene plates using a cheesecloth and was spread homogeneously. Then plates were filled in a dry air sterilizer at 30°C for 48 h. The films (about 0.1 mm thickness) were chilled at room temperature in a desiccator before being manually divided from sterile polyethylene plates.

**TABLE 2:** Sample codes with corresponding compositions of films used throughout the study.

Sample code	Solvent	WPI	OLE
		% (w/v)	% (v/v)
UC	–	–	–
C‐DW	DW	5	0
C‐DW‐OLE	DW	5	5
C‐PAW	PAW	5	0
C‐PAW‐OLE	PAW	5	5

Abbreviations: C, coating; DW, distilled water; OLE, olive leaf extract; PAW, plasma‐activated water; UC, uncoating; WPI, whey protein isolate.

### 2.6 Preparation of cooked meat product

**FIGURE 1:**
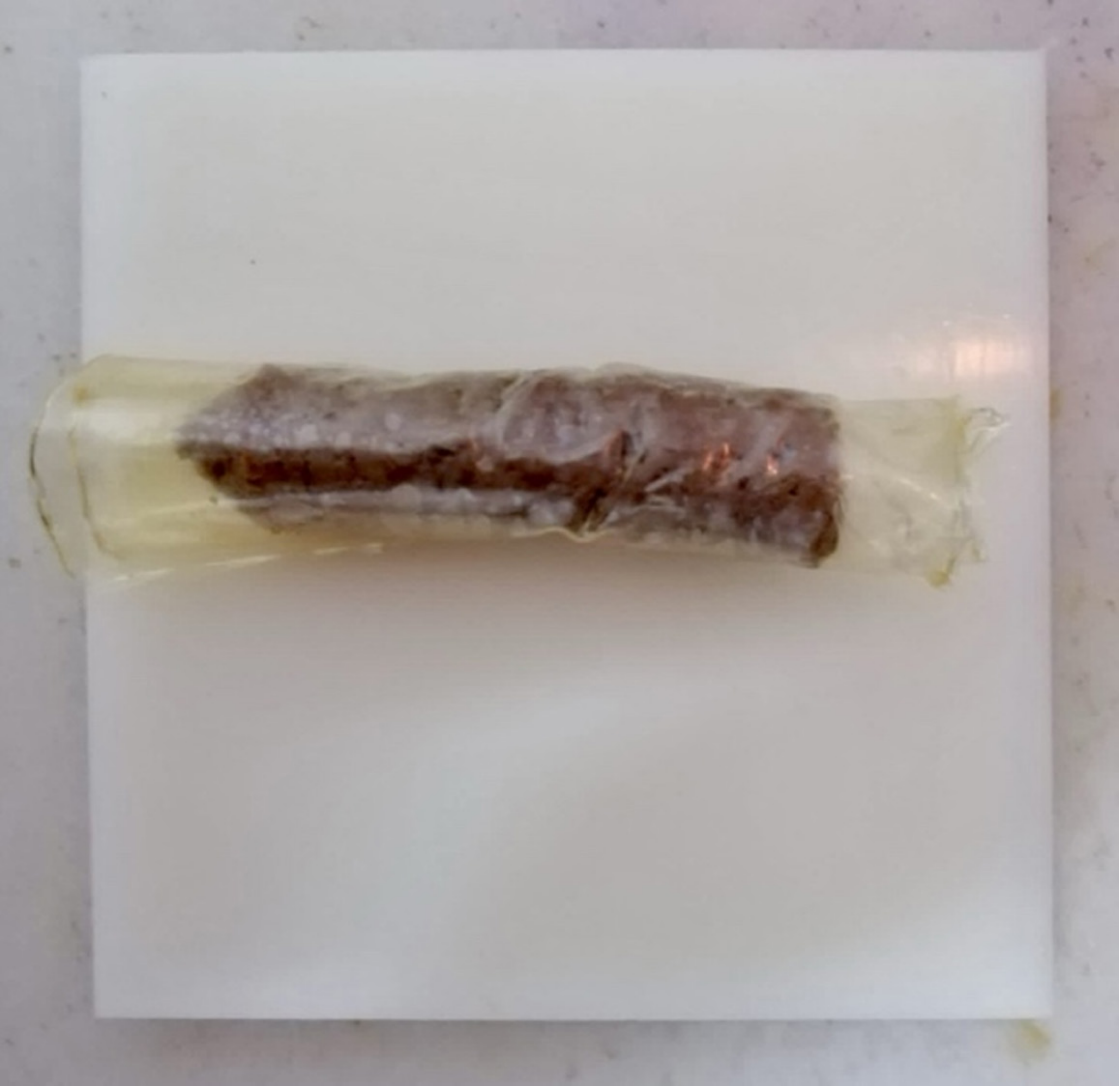
Cooked meat product wrapped with edible film containing PAW and OLE.

Beef (*M. longissimus thoracis* et *lumborum*) was acquired 24 hours after death from a local slaughterhouse (Isparta, TR). The meat was coarsely ground (9.5 mm) and then blended in a mixer before being reground (3.2 mm). The ground meat was given a weight‐based addition of 10% water and 1% sodium chloride. Then put in centrifuge tubes (50 grams each) and heated in a water bath at 60°C. The meat was cooked by heating the water bath to 85°C. The internal cooking temperature was monitored using thermocouples located in the geometric center until it reached 72°C. After cooking, the meat product was chilled at room temperature for 20 min. Then meat (about 40 g) was fully wrapped (Figure 1) with the edible films according to Table 2, and the meat samples without edible film were used as the control. A total of 42 samples were made for each meat and treatment (with and without film). Each sample was placed separately in sterile low‐density polyethylene (LDPE) bags (thick and size of bag were 0.08 mm and 10 × 15 cm, respectively), which were then sealed using an impulse sealer. All samples were stored at 4°C ± 0.5°C for 14 d. pH, CIE L*, a*, b* color values, thiobarbituric acid reactive substances (TBARS), and total mesophilic aerobic bacteria (TMAB), total coliform group bacteria (TCB), mold and yeast counts were carried out on days 0, 1, 7, and 14. Before the analysis, the edible film was removed from the meat, and the exposed outer parts of the samples were discarded, with samples being taken from the middle parts.

### 2.7 Evaluation of shelf life of cooked meat product

#### 2.7.1 pH

The pH values of cooked meat product were measured pH meter (HI 9024, Hanna Instruments, DE) with a penetration spear electrode (FC 200, Hanna Instruments, DE) at 0, 1, 7, and 14 days storage. The pH meter was calibrated with pH 4 and 7 buffer solutions.

#### 2.7.2 Color measurement

The CIE L*, a*, b* color values of cooked meat product were analyzed with a Minolta Colorimeter (CR‐200, D65 Illuminant, Ramsey, USA) at 0, 1, 7, and 14 days storage. The device was configured to measure the CIE L*, a*, b* color scale using an 8.0 mm aperture size, an illuminant 65D, and 10° standard observers. Black and white calibration plates were used for calibration before starting the test. The color value for each sample was measured six times in four replicates, and the results were represented as the mean of the four replicates ± standard error (S.E.).

#### 2.7.3 TBARS

The TBARS values of cooked meat product were analyzed according to Kılıç et al. (2014) at 0, 1, 7, and 14 days of storage. The findings were determined using a standard curve prepared with 1,1,3,3‐tetraethoxypropane and expressed as μmol malondialdehyde (MDA)/kg meat.

#### 2.7.4 Microbiological analysis

The total mesophilic aerobic bacteria (TMAB), total coliform group bacteria (TCB), mold and yeast counts of cooked meat product were evaluated at 0, 1, 7, and 14 days of the storage period. Samples were sterilely weighed 10 g and homogenized by adding 90 mL peptone water. For every sample, serial dilutions were prepared for each sample and the appropriate dilutions were inoculated on the selective medium. Plate Count Agar (Merck, DE), Eosin Methylene Blue Agar (Merck, DE), and Potato Dextrose Agar (Merck, DE) were used for TMAB, TCB, yeast, and molds, respectively.

### 2.8 Statistical analysis

In this study, we prepared five treatments (UC, C‐DW, C‐DW‐OLE, C‐PAW, C‐PAW‐OLE) of cooked meat product in four replicates which were examined during refrigerated storage at 14 d. Analysis of variance (ANOVA) was performed using a general linear model (GLM), with treatment and storage time as fixed effects and their interaction with replicate (batch) as a random variable. After compilation, the results for various quality parameters were analyzed using Jamovi 2.3 software (Sydney, AU). ANOVA and Tukey's multiple range tests were applied to identify significant differences between means at a 1% level (*p* < .001).

## 3 RESULTS AND DISCUSSION

### 3.1 Characterization of PAW composition

In general, reactive oxygen and RONS are often produced when CP and DW interact. It has been observed that these molecules react and dissolve with water molecules, creating a chemical species mixture with low pH and high ORP. Moreover, H_2_O_2_ is generated, which lowers the pH. In this study, the pH of PAW solution was 5.80 (Table 1). It was determined that the 10‐minute plasma treatment decreased the pH of DW by 27.5%. The reduction in pH and the generation of acid‐forming compounds in the PAW can differ depending on the type of plasma (discharge, DBD gliding arc, etc.) (Bruggeman & Leys, 2009), the type of gas (Tian et al., 2015), the power consumed to production PAW, and the exposure time (Rathore et al., 2021).

Nitrogen oxides (NO_x_) are created in air plasma by gas‐phase processes involving dissociated N_2_ and O_2_, which dissolve in water to form NO_2_^−^ and NO_3_^−^. The nitrites’ and nitrates’ concentrations in PAW are presented in Table 1. The concentrations of nitrites and nitrates in PAW can reason cross‐linking of WPI chains, which improves mechanical characteristics (Kramer et al., 2017). Furthermore, heat denaturation of WPI exposes hydrophobic regions and disrupts internal disulfide bonds, thereby allowing intermolecular hydrophobic interactions and cross‐linking of protein chains via disulfide bonds. The resultant films exhibit notably enhanced strength, flexibility, elasticity, and oxygen barrier properties (Brindle & Krochta, 2008).

ORP is thought to be a critical component influencing microbial inactivation because it damages cell membranes and defense systems of microorganisms. Among the ROS generated in PAW, hydrogen peroxide primarily participates in redox reactions, acting either as an oxidizing agent or as a reducing agent (Thirumdas et al., 2018). In this study, it was observed that an enhance in ORP value of DW solution after the CP treatment for 10 min (Table 1). The greater the ORP value, the more likely it is to disrupt both the inner and outer bacteria membranes, causing to cell inactivation (Liao et al., 2007). Some studies demonstrated that the PAW shows remarkable antimicrobial activity in emulsion‐type sausage (Jung et al., 2015), beef (Liao et al., 2020), breast chicken fillets (Sahebkar et al., 2020), and beef jerky (Inguglia et al., 2020).

### 3.2 Total phenolic compounds (TPC) and antioxidant activity of olive leaf extract (OLE)

OLE is an abundant source of phenolic compounds with strong antioxidant activity (Rosa et al., 2019). This effect was also shown by the findings of the current investigation. The amount of TPC in OLE was determined at 71.52 mg GAE/g. This result is in accord with Bilgin and Şahin (2013) reporting a value of 61.66 mg GAE/g in OLE, but higher than the values of 45.42 mg GAE/g and 41.40 mg GAE/g determined by Botsoglou et al. (2014) and Martiny, Pacheco, et al. (2020), respectively. This variability might be attributable to a variety of factors, including the olive leaf collection season, leaf age and cultivar, extraction method, and solvent type (Botsoglou et al., 2014; Brahmi et al., 2012; Elboughdiri, 2018).

The major phenolic compounds in OLE are known to be oleuropein, luteolin, luteolin‐4‐O‐glucoside, luteolin‐7‐O‐glucoside, verbascoside, rutin, hydroxytyrosol, and apigenin‐7‐O‐glucoside (Botsoglou et al., 2014). There are many mechanisms by which phenolic compounds function as antioxidants. Probably the most significant is free radical scavenging, where the phenol can stop a chain reaction of free radicals (Lee & Lee, 2010). The antioxidant activity of OLE was assessed using DPPH and FRAP analyses. By the DPPH test, the IC_50_ value and % antioxidant activity of OLE were determined at 0.46 mg/mL and 77.39%, respectively. This is close to the values of 82.81% reported by Shalaby et al. (2018). However, it is much higher than the value of 49.94% reported in one of the other studies for the antioxidant activity of OLE (Brahmi et al., 2012).

By the FRAP test, the antioxidant activity of OLE was determined at 2.14 mmol Fe^+2^/L. No information could be found in the scientific literature about the Fe^+2^ chelating ability of OLE.

### 3.3 pH

The changes in pH values of cooked meat product are presented in Table 3. On day 0, sample pH values varied from 5.69 to 5.79, but by day 14, values decreased to 5.59 and 5.63. The pH values of whole samples were significantly decreased (*p* < .001) during the storage period. This may be attributable to the impact of the meat's microbiota; proteins were progressively degraded by microorganisms converting organic acids, causing a slight pH drop (Andres‐Bello et al., 2013; Emiroğlu et al., 2010). Similar approach was also stated by Qian et al. (2022) on raw beef patties (formulated with protein‐based edible films containing plasma‐modified essential oil) during 4 d of storage. Moutiq et al. (2020) also determined the in‐package decontamination of chicken breast in‐package disinfection with CP and reported a similar decrease.

**TABLE 3:** pH values of cooked meat product during refrigerated storage.

Samples	Storage time (days)			
	0	1	7	14
UC	5.69 ± 0.00^dB^	5.89 ± 0.01^aA^	5.68 ± 0.01^bBC^	5.67 ± 0.00^aC^
C‐DW	5.79 ± 0.01^aA^	5.78 ± 0.00^bA^	5.67 ± 0.00^bB^	5.63 ± 0.00^bC^
C‐DW‐OLE	5.74 ± 0.00^bA^	5.72 ± 0.00^cAB^	5.71 ± 0.01^aB^	5.61 ± 0.00^cC^
C‐PAW	5.70 ± 0.00^dA^	5.70 ± 0.01^cAB^	5.68 ± 0.00^bB^	5.63 ± 0.01^bC^
C‐PAW‐OLE	5.72 ± 0.01^cA^	5.64 ± 0.01^dB^	5.64 ± 0.01^cB^	5.59 ± 0.01^dC^
	**Interaction (storage time × treatment)**			
	*p*	*F*	*η* ^2^	$\eta_{p}^{2}$
	<.001	388	0.308	0.991

*Note*: Values represent the mean of four replicates ± standard error (SE). Means with different superscripts in a column wise (lower case alphabet) and row wise (upper case alphabet) differ significantly (*p* < .001).

Abbreviations: C, coating; DW, distilled water; OLE, olive leaf extract; PAW, plasma‐activated water; UC, uncoating.

On day 0, the group of C‐DW had the highest (*p* < .001) pH value, while UC and C‐PAW groups had the lowest (*p* < .001) pH values. However, the findings revealed that the pH of UC group enhanced slight after manufactured day, which was attributed to the generation by spoilage microorganisms of basic compounds (Chaijan et al., 2022).

After 14 d storage, the differences in pH among groups varied. The results showed that the UC group had higher (*p* < .001) pH value than the other groups on day 14. It was determined that the C‐DW and C‐PAW groups had similar pH values among themselves. This result may be concluded that CP applied 10 min to the DW was insufficient. In some studies, no change in the pH of meat samples was found even after plasma treatment, for example, for bacon (Kim et al., 2011), cooked meat batter (Jung et al., 2017), and beef loin (Bauer et al., 2017). Such variations are most likely related to plasma reactor type, feed gas utilized in the production of plasma, surface moisture content, and muscle type buffering capacity (Akhtar et al., 2022; Moutiq et al., 2020; Tian et al., 2015). However, the pH value of the C‐DW‐OLE group was lower (*p* < .001) than the pH value of C‐DW group. Similarly, the pH value of C‐PAW‐OLE group was lower (*p* < .001) than the C‐PAW group. These results indicated that the addition of OLE to edible films decreased the pH value of meat, and this could depend on the acidic pH of the extract (Alirezalu et al., 2017). This effect was also observed by Shalaby et al. (2018) in the minced beef produced with irradiated OLE. Also, pH value of the C‐PAW‐OLE group was less (*p* < .001) than the C‐DW‐OLE group, indicating that the usage of C‐PAW in edible film with the addition of OLE reduced pH in cooked meat product. These findings suggested that the C‐PAW with OLE had an interaction effect on the meat's pH.

The antimicrobial components such as oleuropein from OLE and the oxygen barrier from edible film, as well as ROS activity in PAW, can help to maintain pH by inactivating microorganisms that produce basic or acidic compounds, as well as aerobic bacteria. As a result, we can infer that the utilization of PAW in combination with edible film containing OLE helped to preserve the quality of cooked meat product during refrigerated storage by controlling the pH.

### 3.4 Color measurement

Color changes in cooked meat product during the storage period are exhibited in Table 4. The L* values of experimental groups significantly decreased (*p* < .001) during the storage. This reduction in L* values might be due to deterioration induced by microorganisms (Moutiq et al., 2020). As demonstrated in Table 4, no significant difference (*p* > .001) in the L* values of the groups is at the end of the storage. Qian et al. (2022) also observed that no significant variation in L* values of beef patties formulated with protein‐based edible films containing plasma‐modified essential oil. In some studies, it has been also reported that CP treatment did not influence the L* values, for example, for pork or beef (Jayasena et al., 2015), cooked meat batter (Jung et al., 2017), and raw chicken fillets (Wang et al., 2018). The interaction between liquid and plasma causes the liquid to become acidic, resulting in RONS and the formation of reactive oxygen such as NO_2_^−^ and NO_3_^−^ (Oehmigen et al., 2010). Our results suggest that nitrite had no impact on L* values in cooked meat product.

**TABLE 4:** Color values of cooked meat product during refrigerated storage.

Samples	Storage time (days)			
L* values				
	0	1	7	14
UC	59.44 ± 0.47^aB^	64.04 ± 0.14^aA^	58.28 ± 0.55^aBC^	55.59 ± 0.15^aC^
C‐DW	58.54 ± 0.75^aAB^	60.58 ± 0.44^bA^	55.75 ± 0.13^abBC^	53.57 ± 0.43^aC^
C‐DW‐OLE	59.18 ± 0.26^aB^	63.11 ± 0.08^aA^	55.03 ± 0.21^bcC^	53.53 ± 0.35^aC^
C‐PAW	61.10 ± 0.19^aB^	64.16 ± 0.16^aA^	53.80 ± 0.22^bcC^	54.39 ± 0.53^aC^
C‐PAW‐OLE	61.52 ± 0.54^aA^	62.81 ± 0.24^abA^	52.87 ± 0.10^cB^	55.17 ± 0.38^aB^
	**Interaction (storage time × treatment)**			
	*p*	*F*	*η* ^2^	$\eta_{p}^{2}$
	<.001	16.4	0.086	0.814
a* values				
	**0**	**1**	**7**	**14**
UC	11.97 ± 0.16^abB^	12.54 ± 0.28^aB^	14.50 ± 0.21^aAB^	17.07 ± 0.62^aA^
C‐DW	12.20 ± 0.56^abA^	12.42 ± 0.22^abA^	13.86 ± 0.38^aA^	13.87 ± 0.15^bcA^
C‐DW‐OLE	15.06 ± 0.34^aA^	10.98 ± 0.09^abB^	14.83 ± 0.24^aA^	14.16 ± 0.27^abcA^
C‐PAW	12.93 ± 0.29^abBC^	10.99 ± 0.27^abC^	15.72 ± 0.09^aAB^	16.37 ± 0.07^abA^
C‐PAW‐OLE	11.12 ± 0.46^bB^	10.63 ± 0.11^bB^	14.44 ± 0.29^aA^	13.07 ± 0.30^cAB^
	**Interaction (storage time × treatment)**			
	*p*	*F*	*η* ^2^	$\eta_{p}^{2}$
	<.001	13.5	0.241	0.783
b* values				
	**0**	**1**	**7**	**14**
UC	4.46 ± 0.18^aA^	2.12 ± 0.25^aB^	3.24 ± 0.18^aAB^	2.13 ± 0.37^bB^
C‐DW	4.38 ± 0.20^aA^	1.40 ± 0.29^aB^	2.99 ± 0.10^aA^	3.28 ± 0.14^abA^
C‐DW‐OLE	2.10 ± 0.38^bA^	2.87 ± 0.10^aA^	3.70 ± 0.09^aA^	3.57 ± 0.27^abA^
C‐PAW	2.36 ± 0.07^bA^	2.69 ± 0.18^aA^	2.59 ± 0.07^aA^	2.37 ± 0.07^abA^
C‐PAW‐OLE	2.39 ± 0.13^bB^	2.51 ± 0.12^aB^	3.66 ± 0.21^aAB^	4.24 ± 0.19^aA^
	**Interaction (storage time × treatment)**			
	*p*	*F*	*η* ^2^	$\eta_{p}^{2}$
	<.001	21.9	0.605	0.854

*Note*: Values represent the mean of four replicates ± standard error (SE). Means with different superscripts in a column wise (lower case alphabet) and row wise (upper case alphabet) differ significantly (*p* < .001).

Abbreviations: DW, distilled water; OLE, olive leaf extract; PAW, plasma‐activated water.

Throughout the storage period, the a* values of the whole groups mostly remained stable, and no significant modifications of these values were detected. Only UC and C‐PAW groups showed significant changes (*p* < .001) in a* values during storage. UC group had a higher (*p* < .001) a* value than the C‐DW and C‐PAW‐OLE groups at the end of the storage. Also, the results revealed that the a* value of C‐PAW group was higher compared with the C‐DW group; nonetheless, this value was not significant (*p* > .001). Although the use of PAW in the edible film solution tried to slightly increase the a* values, these values decreased with the addition of OLE to the solution. Our approach consistent with other studies (Difonzo et al., 2022; Kurt & Ve Ceylan, 2017), which found a decrease in a* values with the inclusion of OLE into the meat products. According to Alirezalu et al. (2017), adding OLE to frankfurter‐type sausage affects color stability by lowering the a* value. Moreover, the results showed that a* values of C‐DW‐OLE group were found to be similar with the C‐PAW‐OLE group. These findings revealed that the use of PAW in edible film had no significant effect on the a* values of cooked meat product. Likewise, Huang et al. (2019) found that plasma treatment had no impact on the a* values of pork. Furthermore, some studies have been reported a change in a* values in meat samples exposed to plasma (Jayasena et al., 2015; Jung et al., 2017). Such variations are most likely due to the plasma applied directly to the product, as well as the plasma chemistry connected with the discharge systems employed and their operating settings (Moutiq et al., 2020).

The data of b* values were generally no significant changes observed over 14 days in all cooked meat products (*p* > .001). However, the UC group decreased the b* values, while the C‐PAW‐OLE group increased these values during the storage period (*p* < .001). This enhance in b* values might be ascribed to the presence of phenolic compounds in the OLE, which may absorb light at short wavelengths. Shojaee‐Aliabadi et al. (2014) observed a similar effect with carrageenan films containing essential oils. Considering b* values on day 14, no significant (*p* > .001) changes between groups, only whereas the b* value of C‐PAW‐OLE group was higher (*p* < .001) than the UC group. In general, the findings revealed that the use of PAW in EF did not cause a variation in the b* values.

Overall, it is obvious that using plasma‐activated water in edible film did not significantly alter the color of the cooked meat product. However, from these data, it was seen that OLE addition to edible film solution prepared with PAW‐induced more greenness (decrease of a*) and yellowness (increase of b*) in the cooked meat product. Albertos et al. (2017) stated that the olive leaf gelatin films decreased a* and increased b* values with increasing OLE concentration in cold‐smoked Salmon.

### 3.5 TBARS

TBARS values of whole samples enhanced significantly (*p* < .001) during the storage period (Table 5). TBARS values are likely to rise in a high‐oxygen packing environment and over extended storage durations (Ulbin‐Figlewicz & Jarmoluk, 2016).

**TABLE 5:** TBARS (μmol of malondialdehyde/kg meat) values of cooked meat product during refrigerated storage.

Samples	Storage time (days)			
	0	1	7	14
UC	2.50 ± 0.11^aD^	8.18 ± 0.59^aC^	29.27 ± 0.76^aB^	36.29 ± 0.71^aA^
C‐DW	2.61 ± 0.16^aD^	6.20 ± 0.56^abC^	22.23 ± 1.30^bB^	28.70 ± 1.29^bA^
C‐DW‐OLE	2.83 ± 0.12^aB^	3.22 ± 0.23^bAB^	6.66 ± 0.28^cAB^	9.18 ± 0.27^cA^
C‐PAW	2.87 ± 0.14^aD^	6.01 ± 0.45^abC^	18.09 ± 0.96^bB^	24.33 ± 0.80^bA^
C‐PAW‐OLE	2.62 ± 0.24^aC^	4.58 ± 0.41^abBC^	7.69 ± 0.21^cAB^	12.37 ± 0.27^cA^
	**Interaction (storage time × treatment)**			
	*p*	*F*	*η* ^2^	$\eta_{p}^{2}$
	<.001	79.5	0.175	0.955

*Note*: Values represent the mean of four replicates ± standard error (SE). Means with different superscripts in a column wise (lower case alphabet) and row wise (upper case alphabet) differ significantly (*p* < .001).

Abbreviations: DW, distilled water; OLE, olive leaf extract; PAW, plasma‐activated water.

The results revealed that no significant difference (*p* > .001) in TBARS values across the whole groups on day 0. Similarly, on the first day of storage, there was no significant difference in the TBARS values of all groups except the UC and C‐DW‐OLE groups. The results demonstrated that on day 7, UC group had higher (*p* < .001) TBARS values than the other whole groups. A similar approach was also determined on day 14 of storage. The UC group had the highest (*p* < .001) TBARS values. On the 7th and 14th days of the storage, no significant difference (*p* > .001) was observed in TBARS values when the C‐DW group was compared with the C‐PAW group. The use of EF reduced the TBARS values in cooked meat products, while the PAW added to the EF did not affect these values. CP generates reactive species including free radicals as well as positive and negative ions (Mandal et al., 2018). These free radicals may accelerate the oxidation reaction in high‐fat food products. However, in our study, CP may not have affected TBARS values since it was not applied directly to the cooked meat product. According to Jung et al. (2017), the MDA value of meat batters was unaffected by CP. This outcome is like that of Kim et al. (2011), who found that treating CP had no effect on the lipid oxidation in bacon. However, Jayasena et al. (2015) found that CP treatment of beef loin and pork butt increased the lipid oxidation over time. This distinction might be attributed to a different plasma treatment approach and the short plasma lifetime of ROS (Attri et al., 2015). Several studies have demonstrated that plasma accelerates lipid oxidation in meat (Bae et al., 2015; Rod et al., 2012). In addition, the findings revealed that TBARS values of C‐DW‐OLE and C‐PAW‐OLE groups were lower (*p* < .001) than the TBARS values of C‐DW and C‐PAW groups, respectively, on days 7 and 14. Furthermore, the TBARS values of C‐DW‐OLE and C‐PAW‐OLE groups did not show significant differences. As can be seen from these results, OLE added to the edible film solution reduced TBARS values, whether prepared with DW or PAW. To retard the lipid oxidation in meat treated with CP for decontamination, various additives can be used. OLE can prevent lipid oxidation in meat and meat products due to antioxidant activity (Al‐Rimawi et al., 2017; Botsoglou et al., 2014).

### 3.6 Microbiological analysis

During the storage period TMAB, TCB, yeast, and mold levels were beneath the detection limit (<10 CFU/g) in whole groups (data not shown). Ozvural (2019) found no TMAB growth in chicken nugget treated with or without OLE. According to Hayes et al. (2009), adding OLE (100 or 200 μg/g muscle tissue) did not significantly impact TMAB levels in pork patties during refrigerator storage. Likewise, in the present research, no microbial growth was detected in the cooked meat product during the storage time. These could be due to since during the cooking process, and the geometric center temperature of the meat was raised to 72°C, which is far greater than the thermal death points of microorganisms. It was emphasized in different studies that cooking is an effective method to inhibit microbial growth in meat and meat products and ensure the food safety (Jamwal et al., 2015; Noor et al., 2018).

## 4 CONCLUSION

The results indicated that the application of cold plasma applied to DW decreased the pH value and increased the nitrite/nitrate concentrations and ORP value. The amount of TPC was 71.52 mg GAE/g, IC_50_ value was 0.46 mg/mL, and the antioxidant activity was 77.39% in OLE. Also, this study demonstrated that the addition of OLE to edible film prepared with PAW reduced the pH value of cooked meat product during the 14 d storage. The use of PAW in edible film did not impact the CIE L*, a*, b* color values of cooked meat product; however, the addition of OLE reduced the a* values and increased the b* values. According to TBARS results, the use of edible film prevented the lipid oxidation in cooked meat product. Moreover, OLE added to the edible film reduced TBARS values of cooked ground meat, whether prepared with DW or PAW. During the 14‐d storage period, TMAB, TCB, and yeast and mold counts were below the detection limit in cooked meat product.

The findings of this research showed that the addition of OLE to edible film prepared with PAW might potentially prolong the shelf life of meat products without a negative effect on quality parameters of products. To increase the effectiveness of PAW, it may be appropriate to increase the exposure length and intensity of DW to CP or to combine CP utilizing non‐thermal or other hurdle technologies. More studies need to be done on edible films containing PAW and should be thoroughly examined before use in meat and meat products.

## AUTHOR CONTRIBUTIONS

**Damla Bilecen Şen:** Conceptualization (lead); formal analysis (lead); investigation (lead); methodology (equal); writing – original draft (lead); writing – review and editing (equal). **Ali Güleç:** Methodology (equal); writing – review and editing (equal).

## CONFLICT OF INTEREST STATEMENT

The authors state that they have no conflicts of interest.

## ETHICS STATEMENT

This research does not require ethical approval.

## INFORMED CONSENT

All participants in the research provided written informed consent.

## Data Availability

The data used to support the conclusions of this research are accessible from the corresponding author upon reasonable request.
